# Serum Inflammatory Cytokine Levels Correlate with Hand-Foot-Mouth Disease Severity: A Nested Serial Case-Control Study

**DOI:** 10.1371/journal.pone.0112676

**Published:** 2014-11-12

**Authors:** Guangcai Duan, Haiyan Yang, Lubin Shi, Wumei Sun, Meili Sui, Rongguang Zhang, Xinhong Wang, Fang Wang, Weidong Zhang, Yuanlin Xi, Qingtang Fan

**Affiliations:** 1 Department of Epidemiology, College of Public Health, Zhengzhou University, Zhengzhou, Henan, China; 2 Henan Innovation Center of Molecular Diagnosis and Laboratory Medicine, Xinxiang Medical University, Xinxiang, Henan, China; 3 Henan Center for Disease Control and Prevention, Zhengzhou, Henan, China; 4 Children's Hospital of Zhengzhou City, Zhengzhou, Henan, China; Singapore Immunology Network, Agency for Science, Technology and Research (A*STAR), Singapore

## Abstract

**Background:**

Hand-food-mouth disease (HFMD) cases can be fatal. These cases develop rapidly, and it is important to predict the severity of HFMD from mild to fatal and to identify risk factors for mild HFMD. The objective of this study was to correlate the levels of serum inflammatory cytokines with HFMD severity.

**Methods:**

This study was designed as a nested serial case-control study. The data collected included general information, clinical symptoms and signs, laboratory findings and serum cytokine levels.

**Results:**

The levels of IL-4, IL-6, IL-10, TNF-α and IFN-γ in patients with severe HFMD were significantly higher than in mild patients during the 2nd to 5th day after disease onset. The levels of IL-4, IL-6, IL-10 and IFN-γ increased from the 2nd day to the 4th day and later decreased. The levels of TNF-α were high on the first two days and subsequently decreased. The changes of IL-10, TNF-α and IFN-γ in the controls were similar for all cases. The levels of IL-4, IL-6 and IL-17 in the controls were not significantly different with the progression of HFMD.

**Conclusions:**

Our findings indicate that the IL-4, IL-6, IL-10, TNF-α and IFN-γ levels correlate with HFMD severity.

## Introduction

Hand-foot-mouth disease (HFMD) is caused by enterovirus 71 (EV71) and coxsackievirus (CV) A16, and it continues to be a major public health issue in southeastern Asian countries and regions [1]–[4]. HFMD is typically mild and self-limiting. HFMD is most prevalent in children, almost all of whom recover within 4–6 days [5]. However, some patients may quickly develop very severe HFMD with complications, including encephalitis, encephalomyelitis, meningitis, pulmonary edema, circulatory failure, and other serious complications, even death.

Numerous studies have shed light on the etiology and epidemiology of HFMD, and have helped medical professionals and public health officials worldwide understand this condition. In recent years, several HFMD outbreaks occurred in China, Japan, Taiwan, New Zealand and Singapore [1]–[4], [6]–[9]. From May 2008 to April 2009, the China CDC reported 765,220 HFMD cases, including 4,067 severe cases and 205 deaths, with a total incidence rate of 57.9/100,000. Of these, 81.59% were severe cases and 96.43% of the deaths presented with EV71 infection. More than 80% of severe cases and all deaths occurred in 0–6 years age group, and the deaths were primarily due to severe HFMD with complications [10]. The continuing epidemic of HFMD and the potential for serious complications has evoked the highest level of attention from the Chinese government. In response, the Chinese government listed HFMD as a nationally notifiable disease since May 2008. A screening criterion for severity was also recommended according to the clinical manifestations, particularly for children younger than 3-years old. Currently, tests and biomarkers that prospectively predict the progress of HFMD have yet to be developed. Key laboratory findings as potential indicators of severity are not well known, and in particular, the host immune response during the course of the disease also remains unclear. Previous studies examined cellular cytokines in EV71 patients with different outcomes [11]; however, the dynamic changes of these cytokines during the course of disease were unclear.

Therefore, the objective of this study was to determine the risk factors of severe HFMD and to assess the relationship of T cell-related serum inflammatory cytokine levels, including interleukin (IL)-4, IL-6, IL-10, IL-17, interferon (IFN)-γ and tumor necrosis factor (TNF)-α, with the progression of HFMD and its severity.

## Methods

### Study subjects and design

Based on the nested case-control study postulate, our study was designed as a nested serial case-control study. All subjects from the Zhengzhou children's hospital were identified prospectively and diagnosed based on the Chinese guidelines for HFMD diagnosis and treatment (Chinese Ministry of Public Health, revised in 2010) [12]. A total of 102 patients were selected from the hospitalized children (male 62, female 40) aged 6 years and younger (age range, 6–77 months), diagnosed with HFMD during the study period of April 2012 to November 2012 if they had the onset of at least one of the following features: maculopapular or vesicular rash on the palms and/or soles and vesicles or ulcers in the mouth. They were designated prospectively as cases (65 severe cases) and controls (37 mild cases). All subjects were allocated into 4 case-control series according to their course of disease when they visited the hospital as follows: for the 2nd day, 3rd day, 4th day, and 5th day after HFMD onset, the sample size was 9, 15, 22, 19 in the case groups and 9, 9, 9, 10 in the controls, respectively. At the first hospitalized day, serum samples from all patients were obtained.

The study was reviewed and approved by the Life Sciences and Ethics Committee of Zhengzhou University and the Ethics Committee of the Zhengzhou Children's Hospital. Written informed consent was obtained from each participants parents before enrollment.

### Definitions of cases and controls

The cases were defined as having severe or fatal HFMD [13], with severe neurological symptoms, such as febrile seizures, myoclonic jerks, and leg trembling, and more serious complications, including encephalitis, meningitis, acute flaccid paralysis (AFP), cardio respiratory failure, or death. Controls with mild HFMD were without any serious complications.

### Measurements and data collection

The data were collected using a pre-designed structured questionnaire covering the general information, clinical symptoms and signs, laboratory findings, and other factors that may be associated with or indicate severe and fatal HFMD by trained interviewers through face-to-face interviews with the children's parents or babysitters and/or medical reports.

The serum concentrations of IL-4, IL-6, IL-10, IL-17, IFN-γ and TNF-α were measured by enzyme linked immunosorbent assay (ELISA) kits (Shanghai Joyee Biotehnics, Shanghai, China) according to the manufacturer's instructions. The detection limits of these parameters were consistent with the manufacturer's instructions. The collected stool samples were submitted for virus isolation. The samples were inoculated into human embryonic fibroblast (MRC-5), LLC-MK2, HEp-2, and rhabdomyosarcoma (RD) cell cultures. When the enteroviral cytopathic effect involved more than 50% of the cell monolayer, the cells were scraped and indirect fluorescent-antibody staining with a panenteroviral antibody (Chemicon International, Temecula, CA, USA) was performed to identify the enterovirus. These isolates were subsequently identified as EV71 by reverse-transcriptase PCR and sequencing analysis within the VP-1 region of EV71.

### Statistical analysis

The database software program SPSS v. 13.0 (SPSS Inc., USA) was used for statistical analysis. Differences in the means of the continuous variables were tested using Student's t-test or Kruskal-Wallis test, depending on the validity of the normality assumption and the homogeneity of variance. For categorical variables, the proportions were compared using Fisher's exact test or the Chi-square test. A two-tailed p-value of <0.05 was considered significant. Multivariate analysis was performed to examine the adjusted odds ratio for risk factors using logistic regression. A significance level of <0.05 was used for this study.

## Results

### General characteristics of the participants

During the study period of April 2012 to November 2012, 102 patients with HFMD were identified, with 65 cases and 37 controls, who recovered completely with no deaths. The mean time for mild to severe HFMD was 2.51 days (±1.83 days). The mean age was 23.54 months (±13.00 months), and the male/female ratio was 1.5/1. The mean age of the controls was 22.57 months (±12.93 months), and the male/female ratio was 1.6/1. There was no significant difference between the cases and controls for age and gender (*P*>0.05).

**Figure 1:**
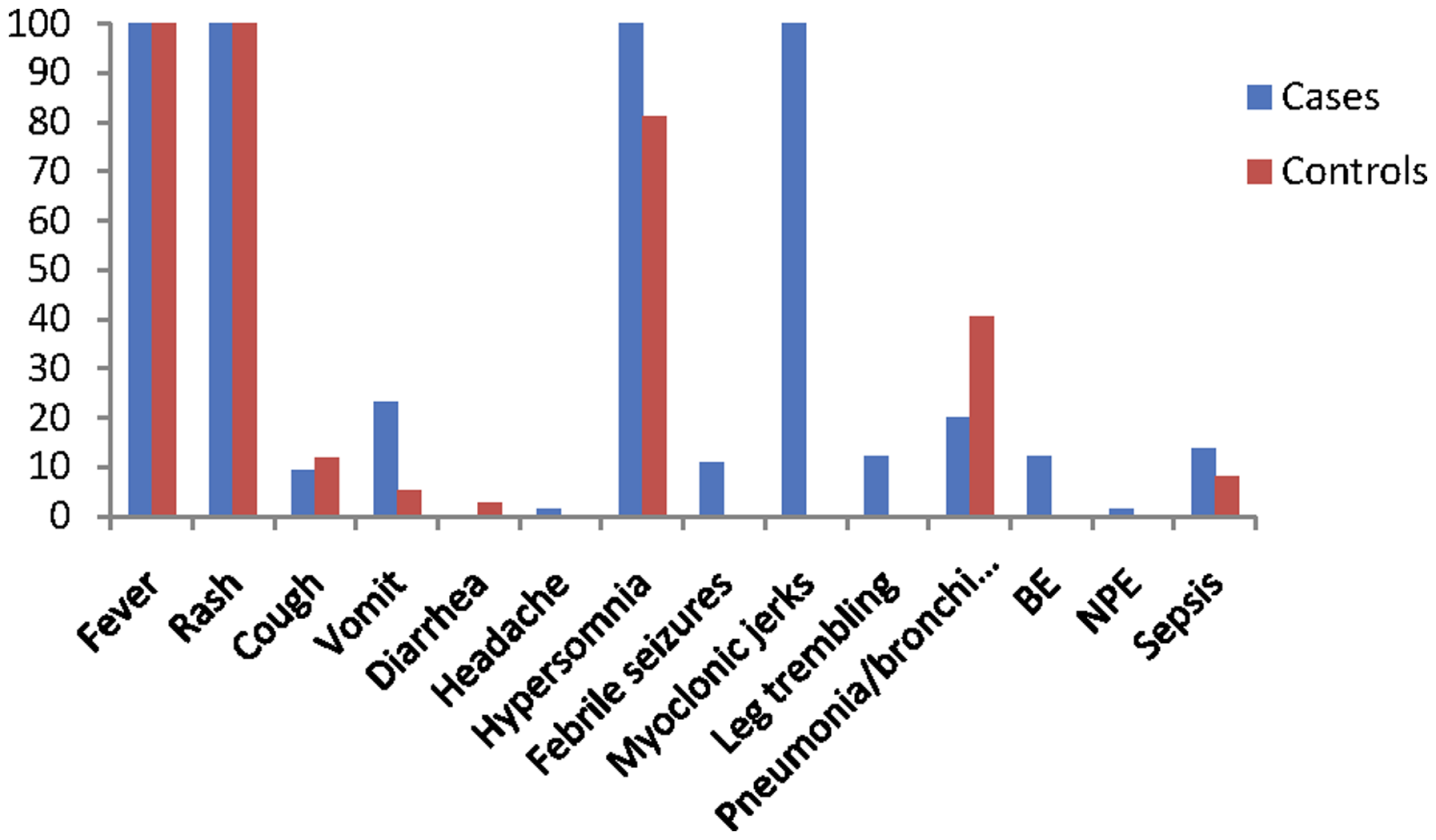
Clinical characteristics of case vs. control patients. BE = brainstem encephalitis; NPE = neurogenic pulmonary edema.

Patient clinical characteristics at hospital admission are presented in Figure 1. Of the 102 patients, the most common symptoms were fever and rash (100% in cases vs. controls), followed by hypersomnia (100% in cases and 81.1% in controls). Neurological symptoms occurred in all cases, including hypersomnia (100%), myoclonic jerks (100%), leg trembling (12.3%) and febrile seizures (10.8%); whereas, no neurological symptoms were present in the controls, except 30 children (81.1%) who presented hypersomnia. Cough occurred in 9.2% cases and 11.8% controls. Of the patients, 23.1% of the cases and 5.4% of the controls were vomiting. Furthermore, of the cases, there were a greater proportion of patients with complications compared to the controls, including brainstem encephalitis (12.3% vs. 0), neurogenic pulmonary edema (1.5% vs. 0), and sepsis (13.8% vs. 8.1). However, the percentage of pneumonia/bronchitis (40.5%) in the controls was higher than in the cases.

### Laboratory findings

Table 1 summarizes the laboratory findings. In the cases vs. controls, laboratory values of white-blood-cell count, neutrophils, lymphocytes, red-blood cell count, hemoglobin, hematocrit, aspartate aminotransferase, acetate dehydrogenase, creatine kinase, α-hydroxybutyric acid, and C-reactive protein levels were not significantly different (*P*>0.05). EV71-positive samples were found in 76.9% of cases vs. 45.9% of controls (*P*<0.05). The degree of fever (>39°C) was 40% vs. 16.2%, duration of fever (>3 days) was 64.9% vs. 18.9, length of in-hospital stay (>5 d) was 53.8% vs. 32.4%, heart rate (>130/min) was 47.7% vs. 32.4%, and respiratory rate (>35/min) was 41.5% vs. 21.6%, for cases and controls, respectively (all *P*<0.05).

**Table 1:** Laboratory values, cases vs. controls.

Variable	Cases (n = 65)	Controls (n = 37)	*P*-value
Degree of fever (>39°C)	26 (40)	6 (16.2)	0.000*
Duration of fever (>3 days)	42 (64.6)	7 (18.9)	0.000*
Length of in-hospital stay (>5 d), n(%)	35 (53.8)	12 (32.4)	0.008*
Heart rate (>130/min), n (%)	31 (47.7)	12 (32.4)	0.005*
Respiratory rate (>35/min), n (%)	27 (41.5)	8 (21.6)	0.000*
WBC×10^9^/l, mean (± SD)	10.44±3.85	10.29±3.23	0.832
PMN percent, mean (± SD)	49.33±16.68	50.49±15.59	0.738
Lymphocytes percent, mean (± SD)	44.39±16.11	41.03±15.04	0.319
RBC×10^12^/l, mean (± SD)	4.35±0.30	4.40±0.33	0.619
Hemoglobin g/l, mean (± SD)	111.48±19.22	106±25.96	0.215
Hematocrit percent, mean (± SD)	44.19±3.72	34.45±2.75	0.185
SGOT U/l, mean (± SD)	34.83±10.08	38.58±20.73	0.242
LDH U/l, mean (± SD)	294.89±43.07	295.67±57.85	0.116
CPK U/l, mean (± SD)	100.19±91.42	77.40±41.01	0.153
α-hydroxybutyric acid U/l, mean (± SD)	199.27±36.44	190.42±45.78	0.100
C-reactive protein mg/l, mean (± SD)	8.98±15.84	11.89±17.33	0.368
IgM g/l, mean (± SD)	1.18±0.35	1.51±2.32	0.075
IgA g/l, mean (± SD)	0.47±0.21	0.49±0.29	0.071
IgG g/l, mean (± SD)	7.26±3.38	6.99±2.16	0.471
C3 g/l, mean (± SD)	1.31±0.27	1.26±0.30	0.806
C4 g/l, mean (± SD)	0.29±0.11	0.35±0.22	0.054
NSE ng/ml, mean (± SD)	22.10±8.66	190.42±45.78	0.485
HEV71 positive, n (%)	50(76.9)	17(45.9)	0.000*

SD, standard deviation; WBC, white blood cells; PMN, polymorphonuclear neutrophils; RBC, red-blood cell; SGOT, serum glutamic oxaloacetic transaminase; LDH, lactate dehydrogenase; CPK, creatine phosphokinase; NSE, neuron-specific enolase.

*Statistically significant difference.

### Multivariate analysis

To determine the relative importance of the factors associated with severity, a multivariate analysis was performed. In the logistic regression model, EV 71-positive (95% CI 1.325–11.550), degree of fever (>39°C) (95% CI 1.088–11.540), duration of fever (>3 days) (95% CI 2.204–19.664), and length of in-hospital stay (>5 d) (95% CI 1.404–12.609) were significantly associated with severity (Table 2).

**Table 2:** Multivariate analysis of the disease factors, cases vs. controls.

Risk factors	β	*SE*	*Waldχ^2^*	*P*	*OR*	95%*CI*
Degree of fever (>39°C)	1.265	0.603	4.408	0.036*	3.543	1.088–11.540
Duration of fever (>3 days)	1.885	0.558	11.395	0.001*	6.584	2.204–19.664
Length of in-hospital stay (>5 d)	1.437	0.560	6.584	0.010*	4.208	1.404–12.609
HEV71 positive	1.364	0.552	6.098	0.014*	3.912	1.325–11.550
Heart rate (>130/min)	0.322	0.624	0.267	0.605	1.380	0.407–4.685
Respiratory rate (>35/min)	0.252	0.707	0.127	0.722	1.286	0.322–5.146

*Statistically significant difference.

### T cell-related serum inflammatory cytokines' levels

**Figure 2:**
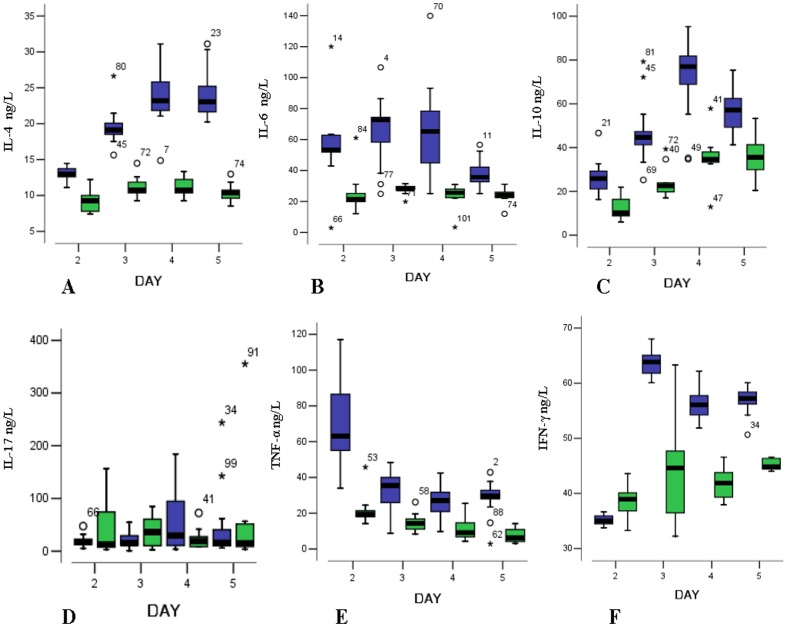
The serum levels of T cell-related immunological cytokines (ng/L, the median, quartiles and extreme values) on the day after HFMD between cases and controls. (A, IL-4; B, IL-6; C, IL-10; D, IL-17; E, TNF-α; F, IFN-γ). The blue box represents cases and the green box represents controls.

Figure 2 indicates the relationships between the levels of T cell-related serum inflammatory cytokines and the course of HFMD onset between cases and controls. The following descriptions for the serum cytokines levels are as the median (P50) and quartiles (P25∼P75). The median serum levels of IL-4 in the cases were significantly higher than in the controls during the 2nd to 5th day after disease onset and were 12.98 (12.60∼13.92) vs. 9.26 (7.61∼11.11), 19.10 (18.32∼20.27) vs. 10.74 (9.81∼12.23), 23.22 (21.74∼26.02) vs. 10.74 (10.00∼12.23), and 23.02 (21.44∼25.42) vs. 10.37 (9.44∼11.02), respectively (*P* = 0.001 for the 2nd day, and *P*<0.001 for the other days). The levels of IL-4 among the cases were significantly different and increased from the 2nd day to 4th day (*P*<0.001). There was no significant difference in the IL-4 levels between the 4th day and 5th day (*P* = 0.564) among cases. The highest level of IL-4 in the cases occurred at the 4th day after disease onset. The controls were not significantly different for HFMD progression (*P* = 0.146). The serum levels of IL-6 of the cases were significantly higher than in the controls during HFMD from days 2 to 5 as follows: 53.22 (47.42∼63.12) vs. 21.40 (16.99∼28.16), 72.49 (56.61∼75.61) vs. 27.97 (26.42∼30.43), 65.20 (44.91∼79.52) vs. 25.71 (22.31∼29.42), and 35.70 (31.46∼43.29) vs. 24.20 (22.31∼26.48), respectively (*P* = 0.019 for the 2nd day and *P*<0.001 for the other days). The levels of IL-6 in the cases were significantly different during the early stage of HFMD (2nd day, 3rd day), and the highest level occurred at 3rd day after disease onset, then significantly decreased from the 4th day (*P*<0.001). The levels of IL-6 among the 4 subgroups in the controls were not significantly different (*P* = 0.148). The levels of IL-10 among the cases were significantly higher than in the controls during the progression of HFMD from days 2 to 5 as follows: 16.86 (12.48∼27.45) vs. 10.18 (7.42∼16.70), 44.66 (41.33∼47.97) vs. 22.50 (19.77∼29.25), 77.00 (68.93∼82.14) vs. 34.64 (32.97∼39.00), and 57.20 (47.97∼62.42) vs. 35.65 (29.77∼42.99), respectively (*P* = 0.001 for the 2nd day and *P*<0.001 for the other days). The levels of IL-10 in the cases were significantly different and increased during the early stage of HFMD from the 2nd day to the 4th day and were the highest at the 4th day, then significantly decreased on the 5th day (*P*<0.001). The levels of IL-10 in the controls were significantly different from the 2nd day to 5th day (*P*<0.001).

The serum levels of IL-17 of the cases were not significantly different than the controls for the 4 stages and were 16.86 (12.48∼27.45) vs. 12.47 (5.93∼88.29), 15.54 (9.85∼31.87) vs. 36.32 (9.41∼71.86), 29.21 (10.07∼99.70) vs. 18.62 (8.54∼34.56), and 15.98 (9.85∼48.86) vs. 14.45 (8.43∼52.80), respectively (*P* = 0.894, 0.222, 0.338 and 0.613, respectively). In the cases, the levels of IL-17 increased from the 3rd day to the 4th day, and then decreased, although the results were not significantly different (*P* = 0.398). In the controls, the levels of IL-17 increased from the 2nd day to the 3rd day and then decreased; however the difference was not significant (*P* = 0.915).

The levels of TNF-α in the cases were significantly higher than in the controls during the four stages and were 63.03 (53.56∼87.42) vs. 19.42 (17.35∼22.90), 35.52 (25.43∼42.55) vs. 14.37 (10.08∼18.21), 26.98 (20.53∼31.86) vs. 9.06 (6.27∼16.52), and 29.60 (28.02∼33.34) vs. 6.15 (4.02∼11.46), respectively (*P* = 0.003 for the 3rd day and *P*<0.001 for the other days). The highest level of TNF-α in the cases occurred at the 2nd day after disease onset, and then significantly decreased from the 2nd day to 4th day (*P*<0.001), and there was no significant difference between the 4th day and the 5th day (*P* = 0.116). The levels of TNF-α in the controls also significantly decreased from the 2nd day to 5th day (*P*<0.001). The levels of IFN-γ in the cases were significantly higher than in the controls during the 4 days and were 35.02 (34.13∼36.29) vs. 38.93 (35.93∼40.80), 63.83 (61.40∼65.21) vs. 44.63 (36.01∼47.69), 56.10 (54.18∼57.94) vs. 41.91 (39.12∼44.63), and 57.23 (56.22∼58.51) vs. 44.84 (44.36∼46.41), respectively (*P* = 0.017 for the 2nd days and *P*<0.001 for the other days). The levels of IFN-γ of the cases significantly increased during the early stage of HFMD from the 2nd day to the 3rd day and then significantly decreased (*P*<0.001). The levels of IFN-γ in the controls were similar (*P* = 0.008).

## Discussion

It is necessary to predict the occurrence of severe or fatal HFMD and identify risk factors for mild HFMD because fatal cases develop rapidly and progress to sympathetic hyperactivity, pulmonary edema and/or pulmonary hemorrhage, and cardiopulmonary collapse [1], [14]–[16]. To prevent the development of severity and avert death though close monitoring and timely intervention, particularly for children younger than 3-years old, the Chinese government has recommended screening criterion for severity. However, different hosts of the same enterovirus, such as EV71, have different clinical symptoms and outcomes [17].

Numerous studies compared the clinical symptoms and manifestations between severe or fatal HFMD and mild HFMD to identify severe and critical cases of HFMD [10], [18]. Currently, we highlight several important signs for characterizing cases of HFMD as severe. Neurological symptoms occurred in all severe cases, including hypersomnia, myoclonic jerks, and/or vomiting, and/or leg trembling, and/or febrile seizures. Notably, myoclonic jerks (100% in cases and 0 in controls) presented during the early stage of onset of severe HFMD, which is easily identifiable and can be used as an early sign to predict central nervous system (CNS) involvement. Chong et al. reported that vomiting may be a signal indicating CNS involvement [19], whereas Yang et al. showed that leg trembling was associated with severe HFMD [13]. In our study, vomiting occurred in 23.1% of the severe cases and 5.4% of the mild HFMD, and only 12.3% of the severe cases exhibited leg trembling. In addition, the severe cases presented a high proportion of complications for brainstem encephalitis, neurogenic pulmonary edema and sepsis. These results may be attributed to the higher number of EV71 positive cases compared to the controls [13]. Previous studies indicated that EV71 is more likely to cause serious complications than other enteroviruses and usually leads to brainstem encephalitis, neurogenic pulmonary edema, and circulation failure [14]–[16]. We also found that EV71 positive status was a significant risk factor for severity. However, serious complications often presented in the late stage of severity and etiological examination usually requires special equipment and is time-consuming, making this application difficult for general medical practice or for every patient [20]. Therefore, key laboratory findings are more helpful, except for significant clinical manifestations. such as myoclonic jerks.

In our patient population, the degree of fever (>39°C), duration of fever (>3 days) and length of in-hospital stay (>5 d) were risk factors for severity. Heart rate variability, using a simplified 5-minute recording, is also useful for detecting changes of the autonomic nervous system and disease progression [21]. No specific laboratory markers were found for biochemical and EV71-specific humoral immunity, except for EV71 infection as an indicator of severity. The biochemical results may be relevant to the spectrum of EV71 clinical manifestations. EV71 infection patients with pulmonary edema had significantly higher median blood levels of glucose and WBC count than patients without pulmonary edema [22], and had higher total WBC counts, absolute bandform counts, absolute neutrophil counts and platelet levels than those with isolated brainstem encephalitis [23]. In our study, because there was only one individual with pulmonary edema, a further stratified analysis was not conducted.

Cellular rather than humoral immunity is correlated with the clinical outcome of EV71 [11]. In our present study, the serum levels of IL-4, IL-6, IL-10, TNF-α and IFN-γ in severe patients were significantly higher than in mild patients during the 2nd to 5th day after disease onset, which indicated that IL-4, IL-6, IL-10, TNF-α and IFN-γ may be involved in disease severity. Whether the median serum levels detected in this study are the best thresholds to identify different stages of HFMD and severity onset, requires additional studies. IL-6 >70 pg/ml was the best predictor of EV71 encephalitis with pulmonary edema [24]. However, the criteria should be different to predict different complications, such as CNS symptoms. Earlier studies indicated that increased TNF-α and IL-6 secretion was detected after EV infection [24], [25]. Studies suggested that the enhanced expression of IL-1, IL-6, IL-10, IL-13, IFN-γ and TNF-α is associated with life-threatening complications in severe HFMD cases [22]–[24], [26], [27]. A recent study has shown that IL-17 is involved in the pathological process of EV71 infection [28]. In our study participants, there was no difference between cases and controls during the stages of disease. Wei et al. showed that an increase of EV71 epitope-specific Th2 type response might portend a poor prognosis for some HFMD patients [29].

In this study, the changes of the serum levels of IL-4, IL-6, IL-10, TNF-α and IFN-γ in severe patients were different. The levels of IL-4, IL-10 and IFN-γ increased for the first three days and then decreased. High levels of IL-6 and TNF-α were detected in severe patients at disease onset, and then decreased with disease progression.

Our study was designed as a nested series case-control study. The patients were enrolled gradually in different series according to their course of disease when they visited the hospital as follows: 2nd day, 3rd day, 4th day, and 5th day after HFMD onset. The samples of exposure biomarkers (cytokines) were collected, and the patients were then allocated into the case group (severe cases) and control group (mild cases) according to the follow-up situations. Therefore, the cases and controls came from the same original population. The changes or trend of the exposure biomarkers during the different stages of disease were analyzed and compared between cases and controls. The study was population-based and the results were of high credibility. This study describes the natural changes of the serum levels of IL-4, IL-6, IL-10, TNF-α and IFN-γ during the stages of HFMD and it progression. Because of medical ethics, the samples were collected only upon the admission of each patient to the hospital. The cytokine profile before and after immunoglobulin treatment significantly decreased for some proinflammatory cytokines in patients with EV71 [23], [30], [31]. However, there are several limitations in this study. The subjects were only hospitalized HFMD children and non-community patients, and the results were also affected by the clinical complications, such as neurological symptoms, brainstem encephalitis, neurogenic pulmonary edema, and even death.

The IL-4, IL-6, IL-10, TNF-α and IFN-γ levels correlated with the progress of HFMD. The levels of IL-4, IL-6, IL-10 and IFN-γ during the progression to severe HFMD significantly increased from the 2nd day to 4th day and then decreased. The levels of TNF-α were high on the first and second day and then significantly decreased. The peak levels of TNF-α, IL-6 and IL-10 in the cases occurred on the 2nd, 3rd, and 4th day after onset of HFMD, respectively.

## References

[1] Ho M, Chen ER, Hsu KH, Twu SJ, Chen KT, et al (1999) An epidemic of enterovirus 71 infection in Taiwan. Taiwan Enterovirus Epidemic Working Group. N Engl J Med 341: 929–935. 10498487 10.1056/NEJM199909233411301

[2] Ma E, Chan KC, Cheng P, Wong C, Chuang SK (2010) The enterovirus 71 epidemic in 2008–public health implications for Hong Kong. Int J Infect Dis 14: e775–780. 20599410 10.1016/j.ijid.2010.02.2265

[3] Momoki ST (2009) Surveillance of enterovirus infections in Yokohama city from 2004 to 2008. Jpn J Infect Dis 62: 471–473. 19934543

[4] Wan J, Zhu L, Liu H, Cao M, Ding Z, et al (2008) Epidemiological analysis of HFMD infected by EV71 in Fuyang City. Anhui Med J 29: 344–345.

[5] Richardson HB Jr, Leibovitz A (1965) “Hand, Foot, and Mouth Disease” in Children; an Epidemic Associated with Coxsakie Virus a-16. J Pediatr 67: 6–12. 14301560 10.1016/s0022-3476(65)80297-9

[6] Ang LW, Koh BK, Chan KP, Chua LT, James L, et al (2009) Epidemiology and control of hand, foot and mouth disease in Singapore, 2001–2007. Ann Acad Med Singapore 38: 106–112. 19271036

[7] CDC (1998) Deaths among children during an outbreak of hand, foot, and mouth disease–Taiwan, Republic of China, April-July 1998. MMWR Morb Mortal Wkly Rep 47: 629–632. 9704628

[8] Chua KB, Kasri AR (2011) Hand foot and mouth disease due to enterovirus 71 in Malaysia. Virol Sin 26: 221–228. 21847753 10.1007/s12250-011-3195-8 PMC8222466

[9] Duff MF (1968) Hand-foot-and-mouth syndrome in humans: coxackie A10 infections in New Zealand. Br Med J 2: 661–664. 5658411 10.1136/bmj.2.5606.661 PMC1991723

[10] Zhang J, Sun J, Chang Z, Zhang W, Wang Z, et al (2011) Characterization of hand, foot, and mouth disease in China between 2008 and 2009. Biomed Environ Sci 24: 214–221. 21784305 10.3967/0895-3988.2011.03.002

[11] Chang LY, Hsiung CA, Lu CY, Lin TY, Huang FY, et al (2006) Status of cellular rather than humoral immunity is correlated with clinical outcome of enterovirus 71. Pediatr Res 60: 466–471. 16940249 10.1203/01.pdr.0000238247.86041.19 PMC7086547

[12] Chinese Ministry of Public Health (revised in 2010). Available: http://www.moh.gov.cn/publicfiles/business/htmlfiles/mohyzs/s3586/201004/246884.htm. Accessed 201010 Jan 202012.

[13] Yang T, Xu G, Dong H, Ye M, He T (2012) A case-control study of risk factors for severe hand-foot-mouth disease among children in Ningbo, China, 2010–2011. Eur J Pediatr 171: 1359–1364. 22527564 10.1007/s00431-012-1731-7

[14] Huang CC, Liu CC, Chang YC, Chen CY, Wang ST, et al (1999) Neurologic complications in children with enterovirus 71 infection. N Engl J Med 341: 936–942. 10498488 10.1056/NEJM199909233411302

[15] Liu CC, Tseng HW, Wang SM, Wang JR, Su IJ (2000) An outbreak of enterovirus 71 infection in Taiwan, 1998: epidemiologic and clinical manifestations. J Clin Virol 17: 23–30. 10814935 10.1016/s1386-6532(00)00068-8

[16] Wang SM, Liu CC, Tseng HW, Wang JR, Huang CC, et al (1999) Clinical spectrum of enterovirus 71 infection in children in southern Taiwan, with an emphasis on neurological complications. Clin Infect Dis 29: 184–190. 10433583 10.1086/520149

[17] Chang LY, Tsao KC, Hsia SH, Shih SR, Huang CG, et al (2004) Transmission and clinical features of enterovirus 71 infections in household contacts in Taiwan. JAMA 291: 222–227. 14722149 10.1001/jama.291.2.222

[18] Zeng M, Zheng X, Wei R, Zhang N, Zhu K, et al (2013) The cytokine and chemokine profiles in patients with hand, foot and mouth disease of different severities in Shanghai, China, 2010. PLoS Negl Trop Dis 7: e2599. 24367714 10.1371/journal.pntd.0002599 PMC3868519

[19] Chong CY, Chan KP, Shah VA, Ng WY, Lau G, et al (2003) Hand, foot and mouth disease in Singapore: a comparison of fatal and non-fatal cases. Acta Paediatr 92: 1163–1169. 14632332

[20] Ryu WS, Kang B, Hong J, Hwang S, Kim J, et al (2010) Clinical and etiological characteristics of enterovirus 71-related diseases during a recent 2-year period in Korea. J Clin Microbiol 48: 2490–2494. 20463159 10.1128/JCM.02369-09 PMC2897491

[21] Lin MT, Wang JK, Lu FL, Wu ET, Yeh SJ, et al (2006) Heart rate variability monitoring in the detection of central nervous system complications in children with enterovirus infection. J Crit Care 21: 280–286. 16990099 10.1016/j.jcrc.2006.02.005

[22] Lin TY, Hsia SH, Huang YC, Wu CT, Chang LY (2003) Proinflammatory cytokine reactions in enterovirus 71 infections of the central nervous system. Clin Infect Dis 36: 269–274. 12539066 10.1086/345905

[23] Wang SM, Lei HY, Huang KJ, Wu JM, Wang JR, et al (2003) Pathogenesis of enterovirus 71 brainstem encephalitis in pediatric patients: roles of cytokines and cellular immune activation in patients with pulmonary edema. J Infect Dis 188: 564–570. 12898444 10.1086/376998

[24] Lin TY, Chang LY, Huang YC, Hsu KH, Chiu CH, et al (2002) Different proinflammatory reactions in fatal and non-fatal enterovirus 71 infections: implications for early recognition and therapy. Acta Paediatr 91: 632–635. 12162592 10.1080/080352502760069016

[25] Chung YC, Ho MS, Wu JC, Chen WJ, Huang JH, et al (2008) Immunization with virus-like particles of enterovirus 71 elicits potent immune responses and protects mice against lethal challenge. Vaccine 26: 1855–1862. 18329759 10.1016/j.vaccine.2008.01.058

[26] Zheng X, Chen X, Gao Y, Fu M, Chen Y, et al (2013) Elevation of human leukocyte antigen-G expression is associated with the severe encephalitis associated with neurogenic pulmonary edema caused by Enterovirus 71. Clin Exp Med DOI: 10.1007/s10238-10013-10237-10236 10.1007/s10238-013-0237-6 23605689

[27] Zhu D, Zhao XY, Yao Y, Dai FF, He H, et al (2013) A new factor influencing pathogen detection by molecular assay in children with both mild and severe hand, foot, and mouth disease. Diagn Microbiol Infect Dis 76: 162–167. 23535205 10.1016/j.diagmicrobio.2013.02.011 PMC7126308

[28] Chen J, Tong J, Liu H, Liu Y, Su Z, et al (2012) Increased frequency of Th17 cells in the peripheral blood of children infected with enterovirus 71. J Med Virol 84: 763–767. 22431024 10.1002/jmv.23254

[29] Wei R, Xu L, Zhang N, Zhu K, Yang J, et al (2013) Elevated antigen-specific Th2 type response is associated with the poor prognosis of hand, foot and mouth disease. Virus Res 177: 62–65. 23886670 10.1016/j.virusres.2013.07.009

[30] Ooi MH, Wong SC, Podin Y, Akin W, del Sel S, et al (2007) Human enterovirus 71 disease in Sarawak, Malaysia: a prospective clinical, virological, and molecular epidemiological study. Clin Infect Dis 44: 646–656. 17278054 10.1086/511073

[31] Wang SM, Lei HY, Huang MC, Su LY, Lin HC, et al (2006) Modulation of cytokine production by intravenous immunoglobulin in patients with enterovirus 71-associated brainstem encephalitis. J Clin Virol 37: 47–52. 16861032 10.1016/j.jcv.2006.05.009

